# Effects of Life Kinetik–based exercise training on cognitive and motor performance in individuals with intellectual disabilities

**DOI:** 10.3389/fpubh.2026.1830603

**Published:** 2026-05-26

**Authors:** Harun Genç, Barış Karaoğlu, Ebru Ceviz, Ziya Bahadır, Ahmet Yalçınkaya, Mustafa Türkmen, Oğulcan Usuflu

**Affiliations:** 1 School of Physical Education and Sports, Bingöl University, Bingöl, Türkiye; 2 School of Physical Education and Sports, Erciyes University, Kayseri, Türkiye; 3 School of Physical Education and Sports, Kırşehir Ahi Evran University, Kırşehir, Türkiye; 4 School of Physical Education and Sports, Mardin Artuklu University, Mardin, Türkiye; 5 Department of Management and Organization, Vocational School, Rumeli University, İstanbul, Türkiye

**Keywords:** attention, cognitive performance, intellectual disabilities, Life Kinetik, motor coordination

## Abstract

**Background:**

Cognitive and motor functions in individuals with intellectual and developmental disabilities are lower than in the general population. This study examined the effects of Life Kinetik (LK) exercises on motor skills and cognitive functions in adolescents with mild intellectual disabilities (MID).

**Methods:**

In this quasi-experimental study, 17 male students (aged 9–12 years; mean 10.47 ± 1.07) were assigned to an experimental group (EG; *n* = 9) or control group (CG; *n* = 8). The EG participated in LK exercises for 60 min, 3 days per week, over 8 weeks, while the CG attended regular physical education classes. Pre- and post-tests assessed cognitive functions using the Corsi Block Test and the d2 Test of Attention, and motor proficiency using bilateral coordination, balance, and upper-limb coordination subtests of the BOT-2 battery. Data were analyzed with two-way repeated measures ANOVA and Bonferroni-adjusted pairwise comparisons. Statistical significance was set at *p* < 0.05.

**Results:**

Significant time × group interactions were found for bilateral and upper-limb coordination, indicating greater improvement in the EG (*p* < 0.05–0.01). Balance improved over time in both groups, with no significant between-group differences. Visual–spatial memory improved over time (Corsi Block Test), while attention, concentration, and psychomotor speed increased and error rates decreased significantly in the EG (d2 Attention Test, *p* < 0.05).

**Conclusion:**

Life Kinetik exercises appear to enhance motor coordination and cognitive performance in adolescents with MID. However, due to the small sample size, these findings should be interpreted with caution, and further studies with larger samples are needed. Integrative exercise programs may be a valuable non-pharmacological strategy to support motor and cognitive development in this population. Integrative exercise programs may be a valuable non-pharmacological strategy to support motor and cognitive development in this population.

## Introduction

Intellectual disability (ID) is a neurodevelopmental disorder that typically manifests during the developmental period and is characterized by significant limitations in both intellectual functioning and adaptive behavior (1). It arises as a result of hereditary or environmental factors related to an individual’s genetic background, leading to delayed or arrested intellectual development during the prenatal, perinatal, or early childhood periods (2). These classifications are generally associated with the level of support required by the individual and may influence motor development outcomes (3). Individuals with mild intellectual disabilities (MID) typically have intelligence quotient (IQ) scores ranging between 50 and 70 (4).

Children and adolescents with intellectual disabilities are at a significantly higher risk of adverse health outcomes compared to their typically developing peers (5). Motor skill difficulties are common in children with ID and may negatively affect their participation in or performance during activities in school, home, and community settings (6). Moreover, as a defining characteristic of ID, cognitive impairment leads to difficulties in daily living activities such as learning, reasoning, problem solving, communication, social interaction, and self-care skills (7).

Globally, ID affects approximately 1% of the population, with about 85% of cases classified as mild (8). The prevalence is markedly higher in low- and middle-income countries compared to high-income countries and is most commonly observed among children and adolescents (9).

In recent years, the education and development of students with intellectual disability have gained increasing importance in the field of special education (10). The process of learning develops through synaptic plasticity. Hearing, visual, and motor abilities are primary domains influenced by neuroplasticity. Exercise plays an important role in the activation of brain plasticity (11). Life Kinetik (LK) exercises are a training program that has been implemented in many countries and is based on stimulating inter-neuronal connections, promoting the formation of new neural networks, reducing potential symptoms, and enhancing performance by regulating the visual system and concentration (12). LK exercises have numerous positive effects, including providing a sense of psychological relaxation, reducing stress, supporting memory and learning processes, promoting physical and mental development, and reducing error rates in various applications (13).

LK provides a multidimensional training program that simultaneously integrates coordinative, cognitive, and visual tasks (14). Primarily aimed at enhancing mental capacity and mobility, LK consists of enjoyable exercises in which learning and performing challenging movements facilitates the formation of new neural pathways in the brain (15). Moreover, in LK exercises, difficulty levels can be adjusted by the individual; exercises planned from easy to difficult promote versatile brain activity, supporting the development of coordination and motor skills (16). The wide applicability of LK allows it to be implemented with individuals of different ages, genders, and physical–mental capacities (17).

In the current literature, studies investigating the effects of Life Kinetik exercises on motor and cognitive skills are limited, and most have been conducted on adults, typically developing individuals, or athletes (18–21). Experimental studies that systematically examine the cognitive and motor benefits of LK in children and adolescents with MID are very limited (22–25). Moreover, existing studies generally involve designs that focus on a single skill area. This highlights the lack of controlled and comprehensive research evaluating the multidimensional effects of LK exercises in individuals with MID.

Zolghadr et al. demonstrated that game-based exercise interventions improve motor coordination in children with intellectual disabilities. Mullhall et al. (24) reported that school-based physical activity programs are feasible and acceptable, while Maicas-Pérez et al. (23) found that such interventions have positive effects on health and physical fitness. Additionally, Diz et al. (22) emphasized that long-term sport-based interventions are more effective. However, these studies predominantly focus on single physical or functional domains, and there is a limited number of controlled studies addressing cognitive and motor skills simultaneously.

This study aims to systematically investigate the effects of Life Kinetik exercises on motor skills (balance, bilateral and upper extremity coordination) and cognitive functions (attention, visuospatial memory, psychomotor speed, focus, and error monitoring) in adolescents with MID. Additionally, it aims to demonstrate the feasibility of LK exercises and the potential gains they may produce across different motor and cognitive domains. This approach is intended to contribute to the academic literature while also providing guidance for practitioners in the fields of special education and rehabilitation.

In this study, it is hypothesized that Life Kinetik exercises will significantly enhance the cognitive skills of adolescents with MID compared to the control group, promote improvements in motor skills, and lead to greater gains in both cognitive and motor performance relative to the control group.

## Materials and methods

### Study design

This research was designed as a quasi-experimental and applied study with a pretest–posttest control group design and was conducted in accordance with the registered protocol without any deviations. Participants were divided into two groups: an experimental group (EG) and a control group (CG). Participants in the EG underwent Life Kinetik exercises for 8 weeks (Table 1) and also continued their regular school-based physical education (PE) classes once per week during the study period. Prior to the intervention, the experimental group completed a familiarization session in which all Life Kinetik exercises were demonstrated and practiced under the supervision of the instructor to ensure correct understanding and safe execution of the movements. During the intervention, continuous verbal, visual, and physical guidance was provided when necessary. The CG did not participate in any exercise program throughout the study period. Participants in the CG attended regular school-based physical education (PE) classes once per week over the eight-week study period. Each PE session lasted approximately 60 min and included standard activities such as warm-up exercises, basic locomotor and coordination activities, simple ball games, and cool-down exercises. Both groups underwent a pretest before the intervention and a posttest after the intervention. During the experimental process, participants’ demographic characteristics were assessed by measuring body weight, height, and body mass index. The Corsi Block Test and the D2 Attention Test were administered to assess cognitive functions. To assess coordination and balance levels, the bilateral coordination, balance, and manual–upper limb coordination subtests of the Bruininks–Oseretsky Test of Motor Proficiency, Second Edition (BOT-2) battery were used. All measurements and test administrations were conducted in accordance with the study protocol.

**Table 1:** 8-week life kinetic exercise programme.

Week	Day	Exercises	Intensities	Duration
Week 1	Mon	Walking on a straight line; Walking + clapping; Step-by-step knee tap – cross-arm combination. Two-foot jumps on the line; Jump + knee/chest combination; Side-to-side jumps without touching the line. Single-leg side jumps (right, then left); Two-foot jumping + clapping.	Light to moderate (2–6)	60 min
	Wed			
	Fri			
Week 2	Mon	Right/left single-leg side jumps + clapping; Double–single leg combination. Double/single-leg landings with coordinated arm movements. Double/single-leg jumps + clapping; Toss ball – catch with both hands; Catch with right hand.	Light to moderate (2–6)	60 min
	Wed			
	Fri			
Week 3	Mon	Catch ball with left hand; Sequential catches (both–right–left); Toss–clap–catch. Toss–clap–catch with right/left hand; Catch ball behind the back (two hands). Behind-the-back catch (right/left); Catch ball from right with right hand, from left with left hand.	Moderate to vigorous (4–6)	60 min
	Wed			
	Fri			
Week 4	Mon	Turn to instructed direction + catch with specified hand; Bounce ball – catch (two hands); Bounce – catch (right hand). Bounce – catch (left hand); Directional bounce tasks; Flamingo stance + ball catch. Flamingo stance on opposite leg + catch; Toss ball + step forward/back; Repeat on opposite leg.	Moderate to vigorous (4–6)	60 min
	Wed			
	Fri			
Week 5	Mon	Toss ball + right/left foot side movement; Right-foot circular motion + ball task. Left-foot circular motion + ball; Walk on line while circling ball around waist; Under-leg pass (right leg). Under-leg pass (left leg); Waist–right–left leg sequence; Dribble with right hand while walking.	Moderate to vigorous (4–6)	60 min
	Wed			
	Fri			
Week 6	Mon	Dribble with left hand while walking; Two-foot jumps + right/left hand dribble. Right-foot jumps + right/left hand dribble; Left-foot jumps + right-hand dribble. Left-foot jumps + left-hand dribble; Double/single-leg landing + dribbling (right/left).	Moderate to vigorous (4–6)	60 min
	Wed			
	Fri			
Week 7	Mon	Balloon toss–catch + foot pass; Left-foot pass + balloon; Tennis ball toss + foot pass. Toss with right hand – catch with left + foot pass; Toss with left – catch with right; Two-ball parallel toss–catch. Two balls tossed with the same hand; Direction tasks using colored scarves; Change direction according to card color.	Moderate to vigorous (4–6)	60 min
	Wed			
	Fri			
Week 8	Mon	Walk with scarf + direction change + counting; Change direction by card color + scarf rotation; Direction change + ball catch. Card-color direction + catch (both/right/left hand); Walking + toss ball – catch with one hand + counting. Card-color direction + ball catch + counting; Counting in twos; Two-ball parallel toss–catch.	Moderate to vigorous (4–6)	60 min
	Wed			
	Fri			

Ethical approval for this study was obtained from the Bingöl University Health Sciences Scientific Research and Publication Ethics Committee (Meeting Date: 24.03.2025; Meeting No: 04; Decision No: 19). The study was conducted in accordance with the principles of the Declaration of Helsinki. Written informed consent was obtained from the parents or legal guardians of all participants prior to enrollment, and verbal assent was obtained from the children prior to participation.

### Participants

The population of this study consisted of students with special needs attending the Special Education Practice School (Grades I, II, and III) in Bingöl Province in 2025. This institution was selected due to its special education structure, the limited opportunities it provides for physical activity and physical education practices, and its accessibility, which facilitated the conduct of the research process.

The sample was determined using a purposive sampling method and consisted of a total of 17 male students aged 9–12 years, diagnosed with mild intellectual disability, and regularly attending physical education classes at school. All participants were included in the study based on official health and special education assessment reports prepared by the relevant specialists.

Participants were assigned to the EG (*n* = 9) and CG (*n* = 8). The demographic and anthropometric characteristics of the participants are presented in detail in Table 2. The mean age of the EG was 10.22 ± 1.09 years, while that of the CG was 10.75 ± 1.04 years. Height was 1.44 ± 0.04 m in the EG and 1.46 ± 0.05 m in the CG. Body weight was 47.44 ± 2.79 kg in the EG and 44.25 ± 3.92 kg in the CG, while BMI values were 22.74 ± 1.70 kg/m^2^ and 20.64 ± 1.41 kg/m^2^, respectively. The groups were found to have similar characteristics in terms of cognitive level, age, height, body weight, and BMI. These findings indicate that the groups were anthropometrically balanced and homogeneous prior to the intervention, making the study sample appropriate for the research objectives and comparable in terms of pre-intervention criteria.

**Table 2:** Demographic characteristics of the participants.

Variable	Experimental group (*n* = 9) (M ± SD)	Control group (*n* = 8) (M ± SD)	Total (*n* = 17)
Age (years)	10.22 ± 1.09 (9,27–11,22)	10.75 ± 1.04 (9,89–11,43)	**10.47 ± 1.07**
Height (cm)	1.44 ± 0.04 (1,41–1,47)	1.46 ± 0.05 (1,42–1,49)	**1.45 ± 0.04**
Body Weight (kg)	47.44 ± 2.79 (45.01–49,98)	44.25 ± 3.92 (41,65–47,46)	**45.94 ± 3.65**
BMI (kg/m^2^)	22.74 ± 1.70 (21,27–24,30)	20.64 ± 1.41 (19,72–21,93)	**21.75 ± 1.87**

M ± SD, Means±standard deviations; BMI, body mass index. Bold values indicate statistically significant results: *p* < 0.05, *p* < 0.01.

### Participant selection criteria

#### Inclusion criteria

- Diagnosis of mild intellectual disability based on official reports prepared by relevant specialists,
- Age between 9 and 12 years,
- No orthopedic, neurological, or cardiovascular health conditions that would limit participation in physical activity,
- Possession of basic motor proficiency sufficient to perform Life Kinetik exercises,
- Written informed consent provided by both the participant and their guardian,
- Ability to attend exercise and testing sessions regularly.

#### Exclusion criteria

- Additional diagnoses such as moderate or severe intellectual disability, autism spectrum disorder, or cerebral palsy,
- Chronic orthopedic, neurological, or cardiovascular conditions that could compromise exercise safety,
- Participation in a regular exercise program within the past 6 months at a level that could affect motor performance,
- Absenteeism or behavioral issues that would prevent regular participation in testing and intervention sessions,
- Lack of parental consent or unwillingness of the participant to take part in the study.

### Exercise intervention

Participants in the EG attended a total of 24 LK exercise sessions over 8 weeks, conducted three times per week (Monday, Wednesday, and Friday; Table 1). Each session lasted approximately 60 min, during which three different categories of LK exercises were performed. LK exercises have a holistic structure that simultaneously targets cognitive processes and motor coordination, as they require the use of multiple body segments at the same time. Additionally, various sports equipment such as balls, rackets, and colored cards were used during the sessions, and participants were asked to manipulate them by throwing, catching, bouncing, or performing similar actions.

Prior to the intervention, all participants in the EG completed a familiarization session. During this session, the exercises were demonstrated in detail by a certified physical education teacher trained in Life Kinetik methods, and participants were given the opportunity to practice each movement pattern under supervision. This process ensured that participants understood the task requirements and were able to perform the exercises safely and correctly before the start of the intervention.

The sessions were delivered by a certified physical education teacher with specialized training in LK exercises, ensuring proper technique and safety. Intervention fidelity was monitored by a research assistant who observed each session and recorded adherence to the prescribed exercises. Participant adherence was tracked by attendance logs, and any deviations from the planned protocol were noted. Safety was continuously monitored during all sessions, with modifications provided for participants who experienced difficulties or fatigue.

Table 1 provides a detailed week-by-week schedule, including exercises, intensities (rated 2–6 on a light-to-vigorous scale), and duration. This structured approach allows for replication in future studies while maintaining both safety and exercise fidelity.

### Materials

#### Anthropometric measurements

Participants’ height was measured using a stadiometer with 0.01 m precision, and body weight was measured without shoes and in light clothing using an electronic scale with 0.01 kg precision. Body mass index (BMI) was calculated by dividing body weight (kg) by the square of height in meters (kg/m^2^).

#### Assessment of motor skills

It was assessed using the Bilateral Coordination, Upper-Limb Coordination, and Balance subtests of the Bruininks–Oseretsky Test of Motor Proficiency, Second Edition (BOT-2). The second edition of the BOT-2 was chosen for this study due to its more comprehensive, up-to-date content and higher psychometric properties compared to the first edition. The BOT-2 allows for a detailed assessment of both gross and fine motor skills in individuals aged 4–21 years (26). The test content has been modernized, with an increased number of items and updated normative values based on a large and representative sample (27). Furthermore, the subtests allow for a detailed profiling of motor skills, enabling a more precise assessment of motor proficiency in individuals with intellectual disabilities (28). The clarity of the administration guidelines and the ability to detect motor problems even with small developmental differences contribute to the widespread use of the BOT-2 in research and rehabilitation settings. Therefore, in the present study, participants’ improvements in bilateral coordination, balance, and upper-limb coordination were assessed using the BOT-2 subtests.

#### Assessment of short-term visuospatial working memory

Short-term visuospatial memory was assessed using the digital version of the Corsi Block Test, in which participants were asked to reproduce the sequence of blocks highlighted on the screen in the same order (29).

#### D2 attention test

The D2 Attention Test was administered to assess students’ selective and sustained attention. Participants were asked to identify the letter “d” with two marks in each row within 20 s. The 14-row test was completed in a total of 280 s, and the data obtained were evaluated according to the standard D2 scoring criteria (30).

### Statistical analysis method

Statistical analyses were performed using SPSS version 26. The distribution characteristics of the variables were first assessed with the Shapiro–Wilk test, and it was determined that the assumptions for parametric analyses were met for all variables.

To examine the effect of the intervention, differences between pretest and posttest measurements of the experimental and control groups were analyzed using a 2 (group: experimental–control) × 2 (time: pretest–posttest) two-way repeated measures ANOVA, which evaluated both time and group effects simultaneously. In cases of significant interactions, Bonferroni-corrected pairwise comparisons were conducted to identify the source of the differences.

Summary data for motor and cognitive tests were presented as mean ± standard deviation (M ± SD), and a significance level of *p* < 0.05 was adopted for all statistical tests. Additionally, partial eta squared (η^2^) values were reported to assess the effect size of the intervention.

## Results

Within-group and between-group results for the BOT-2 subtests of Bilateral Coordination, Upper-Limb Coordination, and Balance are presented in Table 3. Analyses revealed significant Time × Group interactions for Bilateral Coordination and Upper-Limb Coordination, indicating that the EG showed greater improvements compared to the CG. For Bilateral Coordination, the EG improved from 10.25 ± 2.96 to 13.75 ± 2.71 (+34.1%), whereas the CG decreased slightly (9.63 ± 2.62 to 9.13 ± 2.90, −5.2%). The Time × Group interaction was significant (*F* = 5.58, *p* = 0.032, η^2^ = 0.271), suggesting a large effect of the intervention. For Upper-Limb Coordination, the EG increased from 12.11 ± 6.09 to 16.13 ± 6.76 (+33.2%), while the CG showed only a minor improvement (11.13 ± 5.11 to 11.75 ± 4.27, +5.6%). The Time × Group interaction was significant (*F* = 18.87, *p* = 0.001, η^2^ = 0.557), indicating a very large effect of the intervention. For Balance, both groups improved over time (EG: 20.78 ± 4.15 to 25.44 ± 2.65, +22.5%; CG: 20.13 ± 3.27 to 20.88 ± 4.05, +3.7%), but the Time × Group interaction was not significant (*F* = 0.78, *p* = 0.727, η^2^ = 0.128), suggesting that the intervention did not differentially affect balance performance between groups. Overall, the intervention produced significant improvements in motor coordination, particularly for Bilateral and Upper-Limb Coordination, while balance improvements were similar in both groups. Effect sizes ranged from medium to large (η^2^ = 0.128–0.557), indicating meaningful intervention effects.

**Table 3:** Results of 2-way repeated measures ANOVA comparing pre- and post-tests of experimental and control groups on BOT-2 motor tests.

Test	Group	n	Pre-test Mean ± SD	Post-test Mean ± SD	% change	Time×Group		
						F	*p*	η^2^
Bilateral coordination	Experimental	9	10.25 ± 2.96 (7.77–12.72)	13.75 ± 2.71 (11.48–16.01)	+34.1%	5.58	0.032**	0.271
	Control	8	9.63 ± 2.62 (7.24–11.86)	9.13 ± 2.90 (7.09–12.90)	−5.2%			
Upper-limb coordination	Experimental	9	12.11 ± 6.09 (6.56–17.43)	16.13 ± 6.76 (11.24–21.00)	+33.23%	18.87	0.001**	0.557
	Control	8	11.13 ± 5.11 (7.62–15.03)	11.75 ± 4.27 (8.98–15.68)	+5.62%			
Balance	Experimental	9	20.78 ± 4.15 (17.34–24.65)	25.44 ± 2.65 (22.91–27.33)	+22.46%	0.78	0.727	0.128
	Control	8	20.13 ± 3.27 (17.63–22.36)	20.88 ± 4.05 (18.22–25.10)	+3.73%			

Effect sizes (ES) are reported as partial eta squared (η^2^). 0.01–0.059 indicate small effect, 0.06–0.139 indicate medium effect, and ≥0.14 indicate large effect. Bold values indicate statistically significant results: *p < 0.05, **p < 0.01. % Change = [(Post-test mean – Pre-test mean) / Pre-test mean] × 100.

The results of the Corsi Block Test are presented in Table 4. A significant main effect of time was observed (*F*(1,15) = 5.77, *p* = 0.030, η^2^ = 0.278), indicating that participants improved overall from pretest to posttest. The Time × Group interaction was not significant (F(1,15) = 2.31, *p* = 0.149, η^2^ = 0.133), suggesting that the magnitude of change did not differ statistically between the experimental (EG) and control (CG) groups. The main effect of group was also not significant (F(1,15) = 0.48, *p* = 0.500, η^2^ = 0.031), indicating that the overall Corsi performance of the two groups was similar. Although the Time × Group interaction was not statistically significant, percentage improvement analyses showed a greater increase in the EG (+38.5%) compared to the CG (+8.7%), suggesting that the Life Kinetik intervention may have had a positive, albeit non-significant, effect on visuospatial memory.

**Table 4:** Results of 2-way repeated measures ANOVA comparing pre- and post-tests of experimental and control groups on Corsi block test.

Group	n	Pre-test Mean ± SD	Post-test Mean ± SD	% change	Time×Group		
					F	*p*	η^2^
EG	9	1.44 ± 0.60 (0.66–2.22)	1.88 ± 1.02 (1.05–2.94)	+38.5%	2.31	0.149	0.133
CG	8	1.38 ± 0.52 (0.94–1.80)	1.50 ± 0.53 (1.06–1.94)	+8.70%			

Effect sizes (ES) are reported as partial eta squared (η^2^). 0.01–0.059 indicate small effect, 0.06–0.139 indicate medium effect, and ≥0.14 indicate large effect. *p < 0.05, **p < 0.01. % Change = [(Post-test mean – Pre-test mean) / Pre-test mean] × 100.

Results of the d2 Attention Test subparameters are presented in Table 5. Significant Time × Group interactions were observed for all parameters, indicating that the EG improved more than the CG after the 8-week intervention. For TN, a significant improvement was observed in the EG (+3.3%), whereas the CG remained almost unchanged (+0.13%). The Time × Group interaction was significant (*F* = 5.98, *p* = 0.028, η^2^ = 0.285). For E1, the EG showed a reduction of 5.5% while the CG had a negligible change (−0.36%), with a significant Time × Group interaction (*F* = 5.11, *p* = 0.038, η^2^ = 0.254). For E2, the EG decreased by 13.8% versus 0.64% in the CG, with a significant interaction (*F* = 7.04, *p* = 0.018, η^2^ = 0.319). For Er, EG errors decreased by 7%, while CG errors remained nearly constant (Time × Group: *F* = 5.88, *p* = 0.029, η^2^ = 0.282). For TN-E, EG improved by 4.98%, with no significant change in CG (Time × Group: *F* = 5.94, p = 0.028, η^2^ = 0.284). For CP, EG increased by 6.7%, whereas CG decreased slightly (Time × Group: *F* = 6.11, *p* = 0.026, η^2^ = 0.289). For FR, EG improved by 13.5% with a significant interaction (*F* = 6.94, *p* = 0.019, η^2^ = 0.316). Overall, these results indicate that the Life Kinetik and calisthenics intervention significantly enhanced attention, concentration, psychomotor speed, error control, and reaction skills in the experimental group, while no significant changes were observed in the control group. Effect sizes ranged from medium to large (η^2^ = 0.254–0.319), suggesting meaningful intervention effects.

**Table 5:** Results of 2-way repeated measures ANOVA comparing pre- and post-tests of experimental and control groups on d2 attention test.

Parameter	Group	n	Pre-test Mean ± SD	Post-test Mean ± SD	% change	Time×Group		
						F	*p*	η^2^
TN	EG	9	287.8 ± 53.2 (256.20–336,54)	297.3 ± 53.4 (265.92–345,32)	+3.32%	5.98	0.028*	. 285
	CG	8	312.9 ± 36.0 (267.40–337.48)	313.3 ± 36.7 (268.79–339.42)	+0.13%			
E1	EG	9	90.7 ± 194 (72.64–102.10)	85.7 ± 18.2 (73.73–101.15)	−5.5%	5.11	0.038*	. 254
	CG	8	83.8 ± 15.4 (68.54–96.95)	83.5 ± 17.0 (73.22–99.44)	−0.36%			
E2	EG	9	19.3 ± 4.89 (14.57–23.17)	16.6 ± 4.80 (11.90–20.59)	−13.8%	7.04	0.018*	0.319
	CG	8	20.3 ± 3.68 (17.84–23.49)	20.2 ± 3.04 (17.86–22.58)	−0.64%			
Er	EG	9	110.0 ± 19.2 (90.88–121.61)	102.3 ± 18.2 (83.59–114.40)	−7.0%	5.88	0.029*	0.282
	CG	8	104.1 ± 13.7 (94.75–121.46)	103.5 ± 15.4 (93.76–119.34)	−0.29%			
TN-E	EG	9	160.7 ± 60.3 (127.84–215.65)	168.7 ± 57.4 (143.46–219.53)	+4.98%	5.94	0.028*	0.284
	CG	8	188.8 ± 28.9 (139.67–211.87)	189.4 ± 36.2 (134.84–216.49)	+0.32%			
CP	EG	9	102.4 ± 10.7 (95.12–111.62)	109.3 ± 12.0 (102.76–120.48)	+6.7%	6.11	0.026*	0.289
	CG	8	105.5 ± 10.4 (96.13–112.53)	105.1 ± 11.2 (94.66–112.44)	−0.4%			
FR	EG	9	25.9 ± 1.99 (25.19–27.55)	22.4 ± 1.97 (20.71–23.78)	−13.5%	6.94	0.019*	0.316
	CG	8	24.1 ± 3.51 (21.24–26.53)	23.5 ± 4.00 (20.71–26.61)	−2.5%			

TN, Total Number; El, Omissions; E2, Commissions; Er, Total Errors; TN-E, Psychomotor Speed; CP, Concentration Performance; FR, Fluctuation Rate. Effect sizes (ES) are reported as partial eta squared (η^2^). 0.01–0.059 indicate small effect, 0.06–0.139 indicate medium effect, and ≥0.14 indicate large effect. Bold values indicate statistically significant results: *p < 0.05, **p < 0.01. % Change = [(Post-test mean – Pre-test mean) / Pre-test mean] × 100.

## Discussion

Motor skills reflect an individual’s ability to plan and execute purposeful movements and are fundamental to daily living (31). Adolescents with mild intellectual disabilities (MID) typically exhibit lower motor coordination and difficulties in both fine and gross motor skills compared to typically developing peers (32). Therefore, coordination-based supplementary interventions are recommended alongside regular physical activity (33). Evidence suggests that interventions combining cognitive and motor components may be more effective (34).

In this study, the effects of the Life Kinetik program on motor coordination were examined. The findings indicate significant improvements in upper-limb and bilateral coordination in adolescents with MID. Although balance performance did not show a statistically significant difference between groups, the high percentage improvement in the experimental group suggests a potential positive effect on balance. Despite the small sample size (*n* = 17), post-hoc power analyses (Cohen’s d = 0.65–0.70) indicate that meaningful effects on motor coordination were achieved.

These results align with previous research. Adapted Physical Activity programs (35), physical education interventions (36), and cognitive-motor exercises (37) have demonstrated significant improvements in motor skills in individuals with MID. Physical activity has also been shown to improve motor skills in adolescents with autism spectrum disorder, and multi-component interventions enhance motor integration and attention (38, 39).

Cognitive limitations, particularly in attention and visuospatial working memory, affect learning and daily functioning in MID (40, 41). Life Kinetik exercises, requiring both motor and cognitive engagement, may positively influence these processes. In our study, the Corsi Block Test indicated a trend of improvement in visuospatial working memory in the experimental group, consistent with literature showing that multi-component exercises support working memory (42, 43).

Regarding attention, the eight-week Life Kinetik and calisthenics program significantly improved performance in the experimental group. Increases in TN scores and reductions in error parameters indicate improvements in visual scanning, selective attention, and inhibitory control. These findings support the notion that motor-cognitive interventions can enhance attentional processes, consistent with prior studies (44, 45). Aerobic and resistance-based exercise may further support prefrontal cortex-mediated attention mechanisms (46, 47).

However, the literature does not provide fully consistent evidence regarding cognitive outcomes following working memory and physical activity–based interventions in individuals with intellectual disabilities. A meta-analytic review by Danielsson et al. (48) reported that working memory training generally yields only small overall effects, with visuospatial working memory training often showing negligible improvements. In addition, systematic reviews of physical activity interventions indicate that while motor outcomes are relatively consistent, cognitive outcomes such as working memory, attention, and executive functions are highly variable across studies, with several investigations reporting non-significant effects (39). These inconsistencies suggest that cognitive improvements are highly dependent on intervention design, task complexity, intensity, and individual differences within the MID population.

In contrast to these findings, the present study demonstrated improvements in visuospatial working memory following a motor–cognitive integrated intervention. This discrepancy may indicate that simultaneous engagement of motor coordination and cognitive processing, as required in Life Kinetik training, may produce stronger cognitive stimulation than conventional working memory training alone. Therefore, multimodal interventions may represent a more effective approach for enhancing executive functions in individuals with intellectual disabilities.

Overall, this eight-week Life Kinetik program improved motor coordination, attention, visuospatial working memory, and processing speed in adolescents with MID. The results highlight the feasibility and effectiveness of motor-cognitive integrated approaches as an intervention strategy for individuals with cognitive and motor limitations.

## Conclusion

This study suggests that participation of adolescents with MID in a physical activity program supplemented with attention-enhancing activities may contribute to improvements in visual memory, perception, attention, and motor skills. The findings indicate a potential relationship between physical activity and the interaction of motor and cognitive processes. However, considering the small sample size and study limitations, these results should be interpreted with caution. The findings further suggest that physical activity programs incorporating cognitive stimulation and coordinative demands may be beneficial for improving attention and related cognitive functions in individuals with MID. Future research should include larger samples, longer intervention periods, and more comprehensive cognitive assessments to better evaluate the sustainability and generalizability of these effects.

### Limitations of the study

This study has several limitations. First, the relatively small sample size (*n* = 17) and the inclusion of only male participants within a specific age range limit the statistical power and generalizability of the findings. Recruiting adolescents with mild intellectual disabilities who meet strict inclusion criteria and can adhere to an 8-week structured intervention presents practical and ethical challenges. Therefore, these results should be considered preliminary, and caution is needed when generalizing the findings.

Second, the absence of a control group and the use of a repeated-measures design limit causal inferences. In addition, the short intervention duration and lack of follow-up assessment prevent conclusions about long-term effects. Single-site recruitment and lack of blinding may further restrict external validity and introduce potential bias. Moreover, the outcome measures were limited and may not fully capture the multidimensional nature of motor and cognitive performance.

Despite these limitations, post-hoc power analysis for key outcomes showing significant group × time interactions—including bilateral and upper-limb coordination—indicated moderate statistical power (≈68–72%) at *α* = 0.05, based on observed effect sizes (Cohen’s d = 0.65 and d = 0.70, respectively). These results suggest that the intervention produced meaningful effects on motor coordination despite the small sample. Future studies with larger, more diverse samples and more robust designs are warranted to confirm and extend these findings.

## Data Availability

The original contributions presented in the study are included in the article/supplementary material, further inquiries can be directed to the corresponding author.
